# Distigmasterol-Modified Acylglycerols as New Structured Lipids—Synthesis, Identification and Cytotoxicity

**DOI:** 10.3390/molecules26226837

**Published:** 2021-11-12

**Authors:** Magdalena Rudzińska, Aleksandra Grudniewska, Anna Chojnacka, Witold Gładkowski, Gabriela Maciejewska, Anna Olejnik, Katarzyna Kowalska

**Affiliations:** 1 Faculty of Food Science and Nutrition, Poznan University of Life Sciences, 60-637 Poznań, Poland; anna.olejnik@up.poznan.pl (A.O.); katarzyna.kowalska@up.poznan.pl (K.K.); 2 Department of Chemistry, Wrocław University of Environmental and Life Sciences, 50-375 Wrocław, Poland; aleksandra.grudniewska@upwr.edu.pl (A.G.); anna.chojnacka@upwr.edu.pl (A.C.); witold.gladkowski@upwr.edu.pl (W.G.); 3 Faculty of Chemistry, Wrocław University of Science and Technology, 50-371 Wrocław, Poland; gabriela.maciejewska@pwr.edu.pl

**Keywords:** phytosterols, stigmasterol, acylglycerols, synthesis, identification, cytotoxicity

## Abstract

Plant sterols, also referred as phytosterols, have been known as bioactive compounds which have cholesterol-lowering properties in human blood. It has been established that a diet rich in plant sterols or their esters alleviates cardiovascular diseases (CVD), and also may inhibit breast, colon and lung carcinogenesis. Phytosterols, in their free and esterified forms, are prone to thermo-oxidative degradation, where time and temperature affect the level of degradation. Looking for new derivatives of phytosterols with high thermo-oxidative stability for application in foods, our idea was to obtain novel structured acylglycerols in which two fatty acid parts are replaced by stigmasterol residues. In this work, asymmetric (1,2- and 2,3-) distigmasterol-modified acylglycerols (dStigMAs) were synthesized by the covalent attachment of stigmasterol residues to *sn*-1 and *sn*-2 or *sn*-2 and *sn*-3 positions of 3-palmitoyl-*sn*-glycerol or 1-oleoyl-*sn*-glycerol, respectively, using a succinate or carbonate linker. The chemical structures of the synthesized compounds were identified by NMR, HR-MS, and IR data. Moreover, the cytotoxicity of the obtained compounds was determined. The dStigMAs possessing a carbonate linker showed potent cytotoxicity to cells isolated from the small intestine and colon epithelium and liver, whereas the opposite results were obtained for compounds containing a succinate linker.

## 1. Introduction

Structured lipids can be produced enzymatically or chemically via interesterification, acidolysis, and/or esterification processes from conventional fats/oils to improve their nutritional and functional properties [1,2]. They are available in a number of commercial products and are mainly designed for special nutritional applications, especially to meet the growing need for healthier foods and to prevent obesity, cancer, and cardiovascular disease. Plant sterols, also referred to as phytosterols, have been known since the 1950s as bioactive compounds that lower cholesterol levels in human blood. These compounds, both in their free form and as esters, glycosides, or acyl glycosides, are natural components of nuts, seeds, edible oils, and vegetables. Their total content in plant oils is different, and ranges, for example, from 0.4 mg/g in *Nigella sativa* oil to 13.8 mg/g in crude rice bran oil [3,4]. As functional additives to food products, free phytosterols or their esters (PE) with fatty acids are used. The content of phytostanyl/phytosteryl fatty acid esters in margarine enriched in phytosterols amounted to 9 g of PE/100 g, which was equivalent to 5.3–5.4 g phytosterols/phytostanols per 100 g [5]. Phytosteryl esters are endogenous edible oil components present in the range of 6% to 68% of the total sterol content and they are predominantly found in canola/rapeseed, corn, peanut, avocado, evening primrose, and sunflower oils [6]. Epidemiological studies revealed that a diet rich in plant sterols or their esters has protective properties against cardiovascular diseases (CVD) as well as may inhibit breast, colon, and lung carcinogenesis [7,8,9,10]. 

Phytosterols, in their free and esterified forms, are prone to thermo-oxidative degradation. Phytosterol oxidation products, termed oxyphytosterols, are the main products formed during the heating or storage of phytosterols. These compounds play an important physiological and pathological role in humans. In some reports, their cytotoxicity, as well as pro-atherogenic and pro-inflammatory properties are described [11]. The protective effect of unsaturated fatty acids on the oxidation of sterols has been proposed by Barriuso et al. [12]. The unsaturation level of fatty acid moieties influenced the thermo-oxidative stability of stigmasteryl esters [13]. Published data revealed that enrichment of food products with monounsaturated phytosteryl esters provided the lowest rate of degradation. However, this form of sterol protection is not sufficient and further development of sterol derivatives is required to decrease their thermo-oxidative degradation and eliminate the formation of compounds detrimental to human health. 

In earlier studies, Kłobucki et al. [14] synthesized conjugates of phosphatidylcholine with dehydroepiandrosterone (DHEA) which showed no toxicity to normal cells and exhibited sufficient antiproliferative activity against cancer cells. The authors suggested that these compounds can potentially be used as antitumor drugs without any harmful effects on non-cancer cells. The chemical structure of DHEA and sterols is similar. Their skeleton is composed of four condensed rings A, B, C, and D. They possess an equatorially oriented OH group at C3 in ring A, and a double bond at C5-C6 in ring B. Structural differences are observed in the side chain at C17 of the cyclopentane ring D. 

Looking for new derivatives of phytosterols for application in foods, our idea was to obtain structured acylglycerols in which two fatty acid parts are replaced by stigmasterol residues. In this work, asymmetric (1,2- and 2,3-) distigmasterol-modified acylglycerols (dStigMAs) were synthesized. The cytotoxicity of those new lipids was also determined. 

## 2. Results

### 2.1. Synthesis of dStigMAs

**Figure 1:**
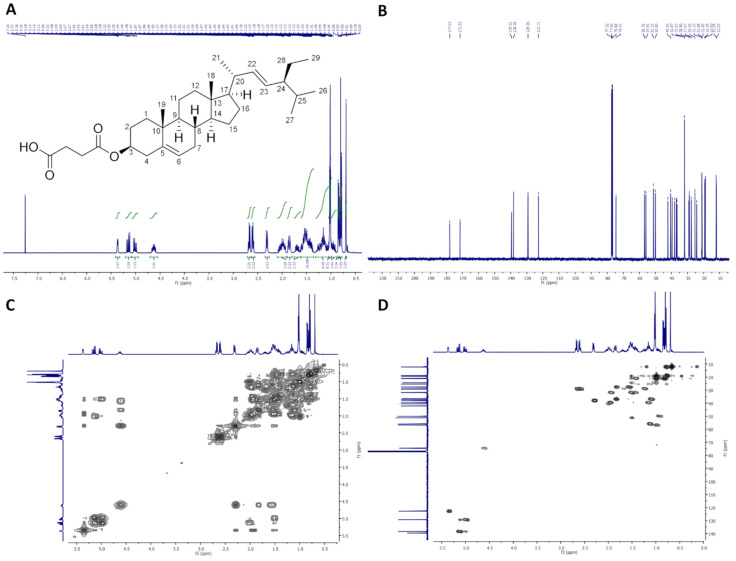
NMR spectra of StigHS: (**A**) ^1^H-NMR (400 MHz, CDCl_3_), (**B**) ^13^C-NMR (100 MHz, CDCl_3_), (**C**) COSY and (**D**) HMQC.

**Figure 2:**
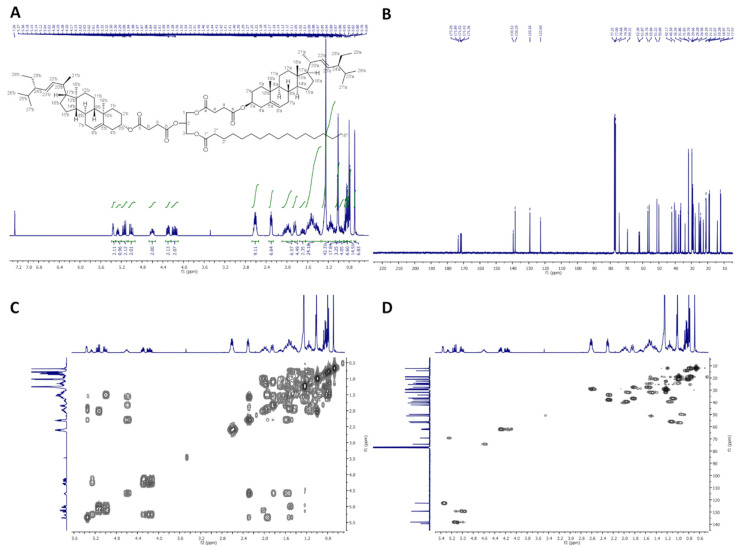
NMR spectra of dStigS-PA: (**A**) ^1^H-NMR (400 MHz, CDCl_3_), (**B**) ^13^C-NMR (100 MHz, CDCl3), (**C**) COSY and (**D**) HMQC.

**Figure 3:**
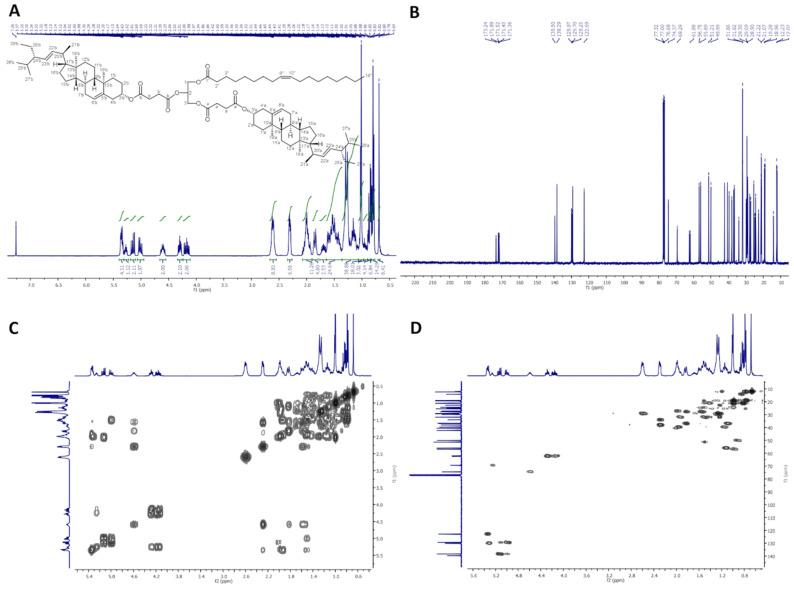
NMR spectra of dStigS-OA: (**A**) ^1^H-NMR (400 MHz, CDCl_3_), (**B**) ^13^C-NMR (100 MHz, CDCl), (**C**) COSY and (**D**) HMQC.

**Figure 4:**
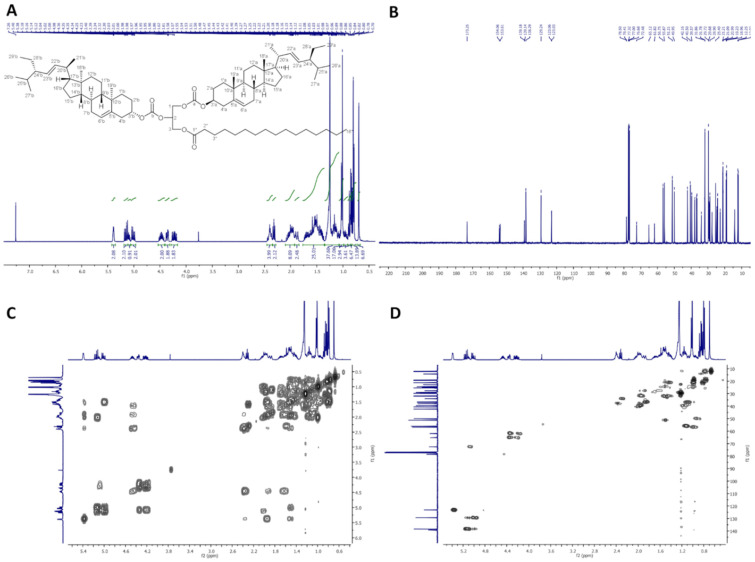
NMR spectra of dStigC-PA: (**A**) ^1^H-NMR (400 MHz, CDCl_3_), (**B**) ^13^C-NMR (100 MHz, CDCl_3_), (**C**) COSY and (**D**) HMQC.

**Figure 5:**
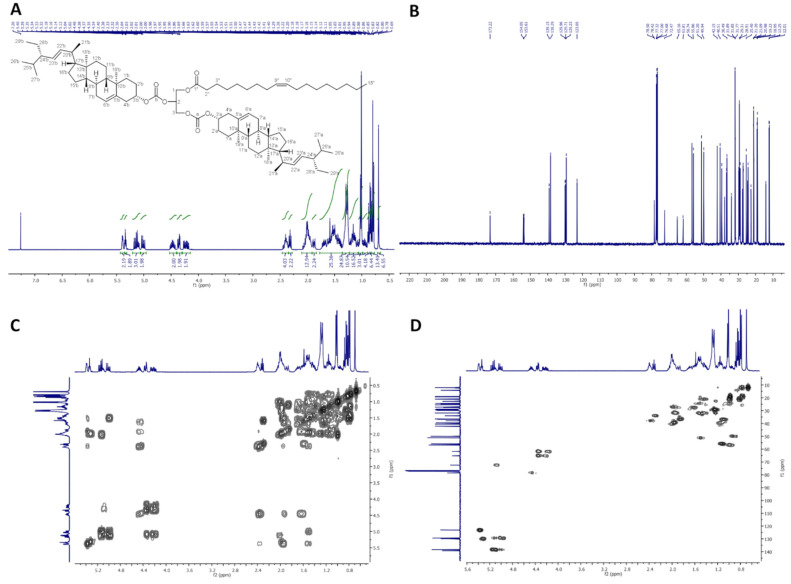
NMR spectra of dStigC-OA: (**A**) ^1^H-NMR (400 MHz, CDCl_3_), (**B**) ^13^C-NMR (100 MHz, CDCl_3_), (**C**) COSY and (**D**) HMQC.

**Scheme 1:**
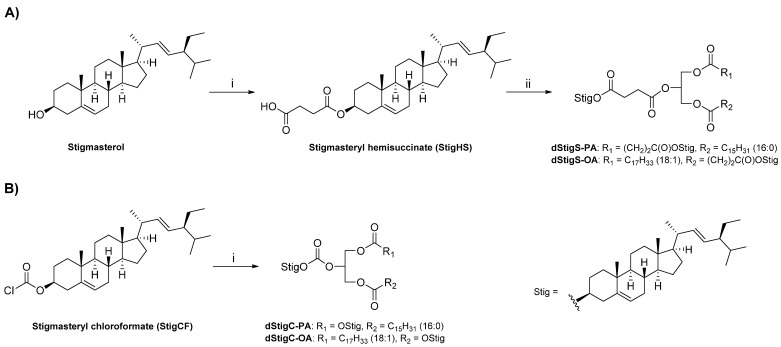
Reagents and conditions: (**A**) (i) succinic anhydride, DMAP, pyridine, 60 °C for 6 h, then 20 °C overnight. (ii) monoacylglycerol (3-palmitoyl-*sn*-glycerol or 1-oleoyl-*sn*-glycerol), DMAP, DCC, CHCl_3_, 20 °C, 24 h. (**B**) (i) monoacylglycerol (3-palmitoyl-*sn*-glycerol or 1-oleoyl-*sn*-glycerol), DMAP, CHCl_3_, 20 °C, 24 h.

The dStigMAs were synthesized by the covalent attachment of stigmasterol residues to *sn*-1 and *sn*-2 or *sn*-2 and *sn*-3 positions of 3-palmitoyl-*sn*-glycerol or 1-oleoyl-*sn*-glycerol, respectively, using a succinate (Scheme 1A) or carbonate linker (Scheme 1B). For the synthesis of acylglycerols containing stigmasterol linked by a succinate residue (dStigS-PA and dStigS-OA), the derivatization of stigmasterol to stigmasteryl hemisuccinate (StigHS) using succinic anhydride in pyridine was required. In the next step, Steglich esterification [15] of the corresponding acyl-*sn*-glycerol with StigHS in the presence of DCC and DMAP afforded the desired acylglycerols in 85–89% yield. For the synthesis of acylglycerols with stigmasterylcarbonoyl residues (dStigC-PA and dStigC-OA), the known reaction of commercially available stigmasteryl chloroformate (StigCF) with corresponding acyl-*sn*-glycerol was applied according to the procedure described in [16] (Scheme 1B) to obtain the final products in 78–94% yield. The details of the synthetic procedures are provided in Section 4.2. The structures of the compounds obtained were determined on the basis of spectroscopic data (Figure 1, Figure 2, Figure 3, Figure 4 and Figure 5).

### 2.2. Yields of Reactions, Physical and Spectroscopic Data

#### 2.2.1. Stigmasteryl Hemisuccinate (StigHS)

On Figure 1, ^1^H-NMR (400 MHz, CDCl_3_), ^13^C-NMR (100 MHz, CDCl_3_), COSY and HMQC spectra of StigHS are presented.

Colorless crystals, m.p. 156–158 °C. Yield, 6.41 g, 86%. TLC: *R*_f_ = 0.47 (CHCl_3_:MeOH:AcOH, 95:5:0.1, *v/v/v*). ^1^H-NMR (400 MHz, CDCl_3_) δ: 0.69 (s, 3H, CH_3_-18), 0.79 (d, *J* = 6.6 Hz, 3H, CH_3_-27), 0.80 (t, *J* = 7.4 Hz, 3H, CH_3_-29), 0.84 (d, *J* = 6.5 Hz, 3H, CH_3_-26), 0.95 (m, 1H, H-9), 0.99–1.06 (m, 2H, H-14 and one of CH_2_-15), 1.02 (s, 3H, CH_3_-19), 1.02 (d, *J* = 6.6 Hz, 3H, CH_3_-21), 1.07–1.31 (m, 5H, one of CH_2_-1, one of CH_2_-12, one of CH_2_-16, H-17 and one of CH_2_-28), 1.36–1.64 (m, 9H, one of CH_2_-2, one of CH_2_-7, H-8, CH_2_-11, one of CH_2_-15, H-24, H-25 and one of CH_2_-28), 1.70 (m, one of CH_2_-16), 1.81–1.89 (m, 2H, one of CH_2_-1 and one of CH_2_-2), 1.92–2.09 (m, 3H, one of CH_2_-7, one of CH_2_-12 and H-20), 2.29–2.34 (m, 2H, CH_2_-4), 2.57–2.63 and 2.65–2.70 (2 × m, 4H, –O(O)C–CH_2_–CH_2_–COOH), 4.63 (m, 1H, H-3), 5.01 (dd, *J* = 15.2 and 8.7 Hz, 1H, H-23), 5.15 (dd, *J* = 15.2 and 8.6 Hz, 1H, H-22), 5.37 (m, 1H, H-6). ^13^C-NMR (100 MHz, CDCl_3_) δ: 12.03 (C-18), 12.24 (C-29), 18.97 (C-27), 19.29 (C-19), 21.00 (C-11), 21.08 (C-26), 21.22 (C-21), 24.34 (C-15), 25.40 (C-28), 27.67 (C-2), 28.90 (C-16), 28.97 and 29.21 (–O(O)C–**C**H_2_–**C**H_2_–COOH), 31.83 (C-7), 31.87 (C-8 and C-25), 36.57 (C-10), 36.93 (C-1), 37.99 (C-4), 39.60 (C-12), 40.50 (C-20), 42.18 (C-13), 50.00 (C-9), 51.22 (C-24), 55.91 (C-17), 56.76 (C-14), 74.53 (C-3), 122.71 (C-6), 129.26 (C-23), 138.30 (C-22), 139.50 (C-5), 171.52 (–O(O)C–), 177.93 (–COOH). IR (ATR) υ_max_ 2937, 2866, 1709, 1440, 1367, 1175, 971 cm^−1^. ESI-HRMS (*m/z*) calcd for C_33_H_52_O_4_ [M + Na]^+^: 535.3763, found: 535.3759.

#### 2.2.2. 1,2-Distigmasterylsuccinoyl-3-Palmitoyl-*sn*-Glycerol (dStigS-PA)

On Figure 2, ^1^H-NMR (400 MHz, CDCl_3_), ^13^C-NMR (100 MHz, CDCl_3_), COSY and HMQC spectra of dStigS-PA are presented.

Colorless crystals, m.p. 91–93 °C. Yield, 1.78 g, 89%. TLC: *R*_f_ = 0.58 (hexane:EtOAc, 3:1, *v/v*). ^1^H-NMR (400 MHz, CDCl_3_) δ: 0.69 (s, 6H, CH_3_-18′ a and b), 0.79 (d, *J* = 6.6 Hz, 6H, CH_3_-27′ a and b), 0.80 (t, *J* = 7.4 Hz, 6H, CH_3_-29′a and b), 0.84 (d, *J* = 6.4 Hz, 6H, CH_3_-26′a and b), 0.87 (t, *J* = 7.0 Hz, 3H, CH_3_-16″), 0.91–0.98 (m, 2H, H-9′ a and b), 0.99–1.06 (m, 4H, H-14′ a and b, and one of CH_2_-15′a and b), 1.01 (s, 6H, CH_3_-19′ a and b), 1.02 (d, *J* = 6.6 Hz, 6H, CH_3_-21′ a and b), 1.07–1.32 (m, 34H, one of CH_2_-1′ a and b, one of CH_2_-12′ a and b, one of CH_2_-16′ a and b, H-17′ a and b, one of CH_2_-28′ a and b, CH_2_-4″, CH_2_-5″, CH_2_-6″, CH_2_-7″, CH_2_-8″, CH_2_-9″, CH_2_-10″, CH_2_-11″, CH_2_-12″, CH_2_-13″, CH_2_-14″, and CH_2_-15″), 1.36–1.64 (m, 20H, one of CH_2_-2′ a and b, one of CH_2_-7′ a and b, H-8′ a and b, CH_2_-11′ a and b, one of CH_2_-15′ a and b, H-24′ a and b, H-25′ a and b, one of CH_2_-28′ a and b, and CH_2_-3″), 1.65–1.75 (m, 2H, one of CH_2_-16′ a and b), 1.81–1.89 (m, 4H, one of CH_2_-1′ a and b and one of CH_2_-2′ a and b), 1.91–2.09 (m, 6H, one of CH_2_-7′ a and b, one of CH_2_-12′ a and b, H-20′ a and b), 2.27–2.34 (m, 6H, CH_2_-4′ a and b, and CH_2_-2″), 2.56–2.67 (m, 8H, –O(O)C–C**H_2_**–C**H_2_**–C(O)O– a and b), 4.15 (dd, *J* = 11.9 and 5.8 Hz, 1H, one of CH_2_-3), 4.19 (dd, *J* = 11.9 and 6.1 Hz, 1H, one of CH_2_-1), 4.29 (dd, *J* = 11.9 and 6.0 Hz, 1H, one of CH_2_-3), 4.30 (dd, *J* = 11.9 and 5.9 Hz, 1H, one of CH_2_-1), 4.55–4.65 (m, 2H, H-3′ a and b), 5.01 (dd, *J* = 15.2 and 8.7 Hz, 2H, H-23′ a and b), 5.15 (dd, *J* = 15.2 and 8.6 Hz, 2H, H-22′ a and b), 5.27 (m, 1H, H-2), 5.34–5.38 (m, 2H, H-6′ a and b). ^13^C-NMR (100 MHz, CDCl_3_) δ: 12.02 (C-18′ a and b), 12.23 (C-29′ a and b), 14.11 (C-16″), 18.97 (C-27′ a and b), 19.28 (C-19′ a and b), 20.99 (C-11′ a and b), 21.07 (C-26′ a and b), 21.22 (C-21′ a and b), 22.68 (C-15″), 24.33 (C-15′ a and b), 24.82 (C-3″), 25.39 (C-28′ a and b), 27.71 (C-2′ a and b), 28.90 (C-16′ a and b), 28.93, 29.09, 29.12, 29.18, 29.27, 29.30, 29.51, 29.70 and 29.75 (–O(O)C–**C**H_2_–**C**H_2_–C(O)O– a and b, C-4″, C-5″, C-6″, C-7″, C-8″, C-9″, C-10″, C-11″, C-12″, C-13″), 31.82 (C-7′ a and b), 31.86 (C-8′ a and b, and C-25′ a and b), 31.91 (C-14″), 33.98 (C-2″), 36.56 (C-10′ a and b), 36.94 (C-1′ a and b), 38.03 (C-4′ a and b), 39.60 (C-12′ a and b), 40.50 (C-20′ a and b), 42.17 (C-13′ a and b), 50.00 (C-9′ a and b), 51.22 (C-24′ a and b), 55.90 (C-17′ a and b), 56.76 (C-14′ a and b), 61.87 (C-3), 62.36 (C-1), 69.31 (C-2), 74.38 (C-3′ a and b), 122.69 (C-6′ a and b), 129.26 (C-23′ a and b), 138.29 (C-22′ a and b), 139.52 (C-5′ a and b), 171.36, 171.43, 171.52 and 171.89 (–O(O)**C**–CH_2_–CH_2_–**C**(O)O– a and b), 173.28 (C-1″). IR (ATR) υ_max_ 2926, 2852, 1732, 1465, 1366, 1154, 972 cm^−1^. ESI-HRMS (*m/z*) calcd for C_85_H_138_O_10_Na [M + Na]^+^: 1342.0188, found: 1342.0178_._

#### 2.2.3. 2,3-Distigmasterylsuccinoyl-1-Oleoyl-*sn*-Glycerol (dStigS-OA)

In Figure 3, ^1^H-NMR (400 MHz, CDCl_3_), ^13^C-NMR (100 MHz, CDCl_3_), COSY, and HMQC spectra of dStigS-OA are presented.

Colorless wax. Yield, 1.60 g, 85%. TLC: *R*_f_ = 0.57 (hexane:EtOAc, 3:1, *v/v*). ^1^H-NMR (400 MHz, CDCl_3_) δ: 0.69 (s, 6H, CH_3_-18′ a and b), 0.79 (d, *J* = 6.6 Hz, 6H, CH_3_-27′ a and b), 0.80 (t, *J* = 7.4 Hz, 6H, CH_3_-29′ a and b), 0.84 (d, *J* = 6.4 Hz, 6H, CH_3_-26′ a and b), 0.88 (t, *J* = 7.0 Hz, 3H, CH_3_-18″), 0.91–0.98 (m, 2H, H-9′ a and b), 0.99–1.06 (m, 4H, H-14′ a and b, and one of CH_2_-15′ a and b), 1.01 (s, 6H, CH_3_-19′ a and b), 1.02 (d, *J* = 6.6 Hz, 6H, CH_3_-21′ a and b), 1.07–1.35 (m, 30H, one of CH_2_-1′ a and b, one of CH_2_-12′ a and b, one of CH_2_-16′ a and b, H-17′ a and b, one of CH_2_-28′ a and b, CH_2_-4″, CH_2_-5″, CH_2_-6″, CH_2_-7″, CH_2_-12″, CH_2_-13″, CH_2_-14″, CH_2_-15″, CH_2_-16″, and CH_2_-17″), 1.36–1.64 (m, 20H, one of CH_2_-2′ a and b, one of CH_2_-7′ a and b, H-8′ a and b, CH_2_-11′ a and b, one of CH_2_-15′ a and b, H-24′ a and b, H-25′ a and b, one of CH_2_-28′ a and b, and CH_2_-3″), 1.65–1.75 (m, 2H, one of CH_2_-16′ a and b), 1.81–1.89 (m, 4H, one of CH_2_-1′ a and b and one of CH_2_-2′ a and b), 1.91–2.09 (m, 10H, one of CH_2_-7′ a and b, one of CH_2_-12′ a and b, H-20′ a and b, CH_2_-8″, and CH_2_-11″), 2.27–2.34 (m, 6H, CH_2_-4′ a and b, and CH_2_-2″), 2.56–2.67 (m, 8H, –O(O)C–C**H_2_**–C**H_2_**–C(O)O– a and b), 4.15 (dd, *J* = 11.9 and 5.8 Hz, 1H, one of CH_2_-3), 4.19 (dd, *J* = 11.9 and 6.2 Hz, 1H, one of CH_2_-1), 4.29 (dd, *J* = 11.9 and 7.4 Hz, 1H, one of CH_2_-3), 4.30 (dd, *J* = 11.9 and 7.1 Hz, 1H, one of CH_2_-1), 4.55–4.65 (m, 2H, H-3′ a and b), 5.01 (dd, *J* = 15.2 and 8.7 Hz, 2H, H-23′ a and b), 5.15 (dd, *J* = 15.2 and 8.6 Hz, 2H, H-22′ a and b), 5.27 (m, 1H, H-2), 5.32–5.38 (m, 4H, H-6′ a and b, H-9″, and H-10″). ^13^C-NMR (100 MHz, CDCl_3_) δ: 12.02 (C-18′ a and b), 12.23 (C-29′ a and b), 14.11 (C-18″), 18.96 (C-27′ a and b), 19.28 (C-19′ a and b), 20.99 (C-11′ a and b), 21.07 (C-26′ a and b), 21.22 (C-21′ a and b), 22.67 (C-17″), 24.33 (C-15′ a and b), 24.79 (C-3″), 25.39 (C-28′ a and b), 27.16 and 27.20 (C-8″ and C-11″), 27.71 (C-2′ a and b), 28.90 (C-16′ a and b), 28.92 and 29.18 (–O(O)C–**C**H_2_–**C**H_2_–C(O)O– a and b), 29.09, 29.12, 29.27, 29.30, 29.51, 29.70 and 29.75 (C-4″, C-5″, C-6″, C-7″, C-12″, C-13″, C-14″ and C-15″), 31.82 (C-7′ a and b), 31.86 (C-8′ a and b, and C-25′ a and b), 31.88 (C-16″), 33.96 (C-2″), 36.56 (C-10′ a and b), 36.93 (C-1′ a and b), 38.03 (C-4′ a and b), 39.60 (C-12′ a and b), 40.50 (C-20′ a and b), 42.17 (C-13′ a and b), 49.99 (C-9′ a and b), 51.21 (C-24′ a and b), 55.89 (C-17′ a and b), 56.75 (C-14′ a and b), 61.88 (C-3), 62.37 (C-1), 69.29 (C-2), 74.37 (C-3′ a and b), 122.69 (C-6′ a and b), 129.25 (C-23′ a and b), 129.70 and 129.97 (C-9″ and C-10″), 138.29 (C-22′ a and b), 139.50 (C-5′ a and b), 171.36, 171.43, 171.52 and 171.89 (–O(O)**C**–CH_2_–CH_2_–**C**(O)O– a and b), 173.24 (C-1″). IR (ATR) υ_max_ 2927, 2852, 1732, 1459, 1377, 1154, 972 cm^−1^. ESI-HRMS (*m/z*) calcd for C_87_H_140_O_10_Na [M + Na]^+^: 1368.0344, found: 1368.0348_._

#### 2.2.4. 1,2-Distigmasterylcarbonoyl-3-Palmitoyl-*sn*-Glycerol (dStigC-PA)

In Figure 4, ^1^H-NMR (400 MHz, CDCl_3_), ^13^C-NMR (100 MHz, CDCl_3_), COSY, and HMQC spectra of dStigC-PA are presented.

Colorless crystals, m.p. 72–74 °C. Yield, 1.72 g, 94%. TLC: *R*_f_ = 0.62 (hexane:EtOAc, 9:1, *v/v*). ^1^H-NMR (400 MHz, CDCl_3_) δ:0.70 (s, 6H, CH_3_-18′ a and b), 0.79 (d, *J* = 6.6 Hz, 6H, CH_3_-27′ a and b), 0.80 (t, *J* = 7.4 Hz, 6H, CH_3_-29′ a and b), 0.84 (d, *J* = 6.4 Hz, 6H, CH_3_-26′ a and b), 0.88 (t, *J* = 7.0 Hz, 3H, CH_3_-16″), 0.91–0.98 (m, 2H, H-9′ a and b), 0.99–1.06 (m, 4H, H-14′ a and b, and one of CH_2_-15′ a and b), 1.01 (s, 6H, CH_3_-19′ a and b), 1.02 (d, *J* = 6.6 Hz, 6H, CH_3_-21′ a and b), 1.07–1.33 (m, 34H, one of CH_2_-1′ a and b, one of CH_2_-12′ a and b, one of CH_2_-16′ a and b, H-17′ a and b, one of CH_2_-28′ a and b, CH_2_-4″, CH_2_-5″, CH_2_-6″, CH_2_-7″, CH_2_-8″, CH_2_-9″, CH_2_-10″, CH_2_-11″, CH_2_-12″, CH_2_-13″, CH_2_-14″, CH_2_-15″), 1.36–1.76 (m, 22H, one of CH_2_-2′ a and b, one of CH_2_-7′ a and b, H-8′ a and b, CH_2_-11′ a and b, one of CH_2_-15′ a and b, one of CH_2_-16′ a and b, H-24′ a and b, H-25′ a and b, one of CH_2_-28′ a and b, CH_2_-3″), 1.84–2.09 (m, 10H, one of CH_2_-1′ a and b, one of CH_2_-2′ a and b, one of CH_2_-7′ a and b, one of CH_2_-12′ a and b, H-20′ a and b), 2.32 (t, *J* = 7.6 Hz, 2H, CH_2_-2″), 2.35–2.45 (m, 4H, CH_2_-4′ a and b), 4.20 (dd, *J* = 12.0 and 5.8 Hz, 1H, one of CH_2_-3), 4.24 (dd, *J* = 11.9 and 6.0 Hz, 1H, one of CH_2_-1), 4.36 (dd, *J* = 12.0 and 4.1 Hz, 1H, one of CH_2_-3), 4.37 (dd, *J* = 11.9 and 4.1 Hz, 1H, one of CH_2_-1), 4.42–4.53 (m, 2H, H-3′ a and b), 5.01 (dd, *J* = 15.2 and 8.7 Hz, 2H, H-23′ a and b), 5.10 (m, 1H, H-2), 5.15 (dd, *J* = 15.2 and 8.6 Hz, 2H, H-22′ a and b), 5.37–5.41 (m, 2H, H-6′ a and b). ^13^C-NMR (100 MHz, CDCl_3_) δ: 12.02 (C-18′ a and b), 12.25 (C-29′ a and b), 14.13 (C-16″), 18.96 (C-27′ a and b), 19.23 (C-19′ a and b), 20.99 (C-11′ a and b), 21.09 (C-26′ a and b), 21.21 (C-21′ a and b), 22.69 (C-15″), 24.32 (C-15′ a and b), 24.80 (C-3″), 25.40 (C-28′ a and b), 27.57 and 27.59 (C-2′ a and b), 28.90 (C-16′ a and b), 29.10, 29.28, 29.37, 29.48, 29.68 and 29.72 (C-4″, C-5″, C-6″, C-7″, C-8″, C-9″, C-10″, C-11″, C-12″, C-13″), 31.78 (C-7′ a and b), 31.86 (C-8′ a and b, and C-25′ a and b), 31.92 (C-14″), 34.00 (C-2″), 36.50 (C-10′ a and b), 36.79 (C-1′ a and b), 37.89 (C-4′ a and b), 39.57 (C-12′ a and b), 40.50 (C-20′ a and b), 42.16 (C-13′ a and b), 49.95 (C-9′ a and b), 51.21 (C-24′ a and b), 55.87 (C-17′ a and b), 56.75 (C-14′ a and b), 61.82 (C-3), 65.12 (C-1), 72.48 (C-2), 78.41 and 78.50 (C-3′ a and b), 123.03 and 123.06 (C-6′ a and b), 129.24 (C-23′ a and b), 138.29 (C-22′ a and b), 139.14 and 139.18 (C-5′ a and b), 153.61 and 154.06 (–OC(O)O– a and b), 173.25 (C-1″). IR (ATR) υ_max_ 2927, 2853, 1739, 1456, 1370, 1243, 1159, 972 cm^−1^. ESI-HRMS (*m/z*) calcd for C_79_H_130_O_8_Na [M + Na]^+^: 1229.9663, found: 1229.9666_._

#### 2.2.5. 2,3-Distigmasterylcarbonoyl-1-Oleoyl-*sn*-Glycerol (dStigC-OA)

In Figure 5, ^1^H-NMR (400 MHz, CDCl_3_), ^13^C-NMR (100 MHz, CDCl_3_), COSY, and HMQC spectra of dStigC-OA are presented.

Colorless wax. Yield, 1.61 g, 78%. TLC: *R*_f_ = 0.62 (hexane:EtOAc, 9:1, *v/v*). ^1^H-NMR (400 MHz, CDCl_3_) δ: 0.69 (s, 6H, CH_3_-18′ a and b), 0.79 (d, *J* = 6.6 Hz, 6H, CH_3_-27′ a and b), 0.80 (t, *J* = 7.4 Hz, 6H, CH_3_-29′ a and b), 0.84 (d, *J* = 6.4 Hz, 6H, CH_3_-26′ a and b), 0.88 (t, *J* = 7.0 Hz, 3H, CH_3_-18″), 0.91–0.98 (m, 2H, H-9′ a and b), 0.99–1.06 (m, 4H, H-14′ a and b, and one of CH_2_-15′ a and b), 1.01 (s, 6H, CH_3_-19′ a and b), 1.02 (d, *J* = 6.6 Hz, 6H, CH_3_-21′ a and b), 1.07–1.33 (m, 30H, one of CH_2_-1′ a and b, one of CH_2_-12′ a and b, one of CH_2_-16′ a and b, H-17′ a and b, one of CH_2_-28′ a and b, CH_2_-4″, CH_2_-5″, CH_2_-6″, CH_2_-7″, CH_2_-12″, CH_2_-13″, CH_2_-14″, CH_2_-15″, CH_2_-16″, CH_2_-17″), 1.36–1.76 (m, 22H, one of CH_2_-2′ a and b, one of CH_2_-7′ a and b, H-8′ a and b, CH_2_-11′ a and b, one of CH_2_-15′ a and b, one of CH_2_-16′ a and b, H-24′ a and b, H-25′ a and b, one of CH_2_-28′ a and b, CH_2_-3″), 1.84–2.09 (m, 14H, one of CH_2_-1′ a and b, one of CH_2_-2′ a and b, one of CH_2_-7′ a and b, one of CH_2_-12′ a and b, H-20′ a and b, CH_2_-8″, CH_2_-11″), 2.32 (t, *J* = 7.6 Hz, 2H, CH_2_-2″), 2.35–2.45 (m, 4H, CH_2_-4′ a and b), 4.20 (dd, *J* = 12.1 and 5.7 Hz, 1H, one of CH_2_-3), 4.25 (dd, *J* = 11.9 and 6.2 Hz, 1H, one of CH_2_-1), 4.33–4.39 (m, 2H, one of CH_2_-1 and one of CH_2_-3), 4.42–4.53 (m, 2H, H-3′ a and b), 5.01 (dd, *J* = 15.2 and 8.7 Hz, 2H, H-23′ a and b), 5.10 (m, 1H, H-2), 5.15 (dd, *J* = 15.2 and 8.6 Hz, 2H, H-22′ a and b), 5.31–5.36 (m, 2H, H-9″ and H-10″), 5.37–5.41 (m, 2H, H-6′ a and b). ^13^C-NMR (100 MHz, CDCl_3_) δ: 12.01 (C-18′ a and b), 12.25 (C-29′ a and b), 14.13 (C-18″), 18.95 (C-27′ a and b), 19.23 (C-19′ a and b), 20.98 (C-11′ a and b), 21.09 (C-26′ a and b), 21.20 (C-21′ a and b), 22.68 (C-17″), 24.32 (C-15′ a and b), 24.76 (C-3″), 25.40 (C-28′ a and b), 27.16 and 27.20 (C-8″ and C-11″), 27.56 and 27.59 (C-2′ a and b), 28.91 (C-16′ a and b), 29.06, 29.09, 29.17, 29.31, 29.52, 29.70 and 29.75 (C-4″, C-5″, C-6″, C-7″, C-12″, C-13″, C-14″, C-15″), 31.77 (C-7′ a and b), 31.85 (C-8′ a and b, and C-25′ a and b), 31.89 (C-16″), 33.96 (C-2″), 36.49 (C-10′ a and b), 36.78 (C-1′ a and b), 37.87 and 37.91 (C-4′ a and b), 39.56 (C-12′ a and b), 40.51 (C-20′ a and b), 42.15 (C-13′ a and b), 49.94 (C-9′ a and b), 51.20 (C-24′ a and b), 55.86 (C-17′ a and b), 56.74 (C-14′ a and b), 61.81 (C-3), 65.16 (C-1), 72.47 (C-2), 78.42 and 78.50 (C-3′ a and b), 123.05 (C-6′ a and b), 129.23 (C-23′ a and b), 129.71 and 129.96 (C-9″ and C-10″), 138.29 (C-22′ a and b), 139.15 (C-5′ a and b), 153.61 and 154.05 (–OC(O)O– a and b), 173.22 (C-1″). IR (ATR) υ_max_ 2928, 2853, 1744, 1459, 1369, 1239, 1162, 972 cm^−1^. ESI-HRMS (*m/z*) calcd for C_81_H_132_O_8_Na [M + Na]^+^: 1255.9819, found: 1255.9822_._

### 2.3. Cytotoxicity

**Figure 6:**
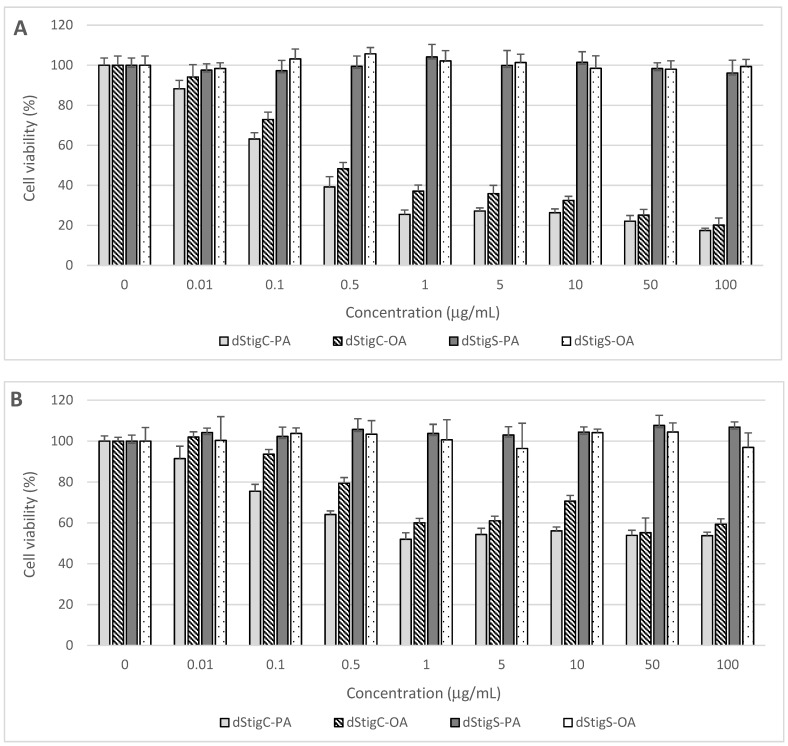

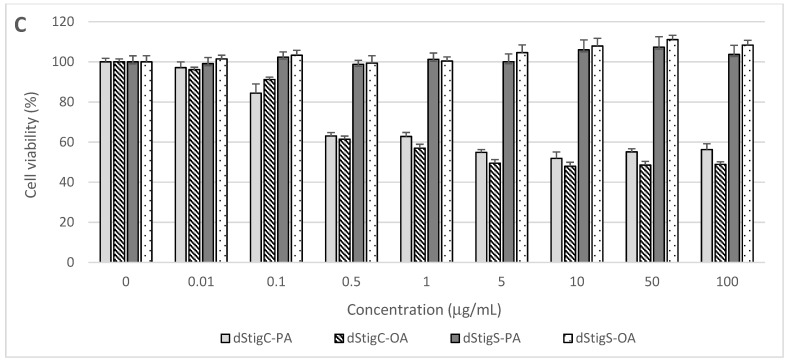
Effect of dStigMAs on (**A**) normal small intestinal epithelial cells (FHs 74 Int, ATCC^®^ CCL241™), (**B**) normal colonic epithelial cells (CCD 841CoN, ATCC^®^ CRL-179™), and (**C**) normal liver cells (THLE-2, ATCC^®^ CRL-2706™).

Distigmasterol-modified acylglycerols were examined for their cytotoxicity in normal human cells originating from the digestive system. The cells were treated for 48 h with increasing concentrations of dStigMAs, and cell proliferation, metabolic activity, and viability were assayed using the MTT test. The cytotoxic effects of the analyzed dStigMAs on CCD 841CoN colon mucosa cells, FHs 74 Int small intestine epithelial cells, and THLE-2 hepatocytes are presented in Figure 6.

Distigmasterol-modified acylglycerols (dStigMAs) possessing a carbonate linker showed potent cytotoxicity to the cells isolated from the small intestine (Figure 6A) and colon (Figure 6B), as well as epithelium and liver (Figure 6C). The highest cytotoxic potential was observed in small intestinal FHs 74 Int cells with the first cytotoxic symptoms noted after their treatment with dStigC-PA and dStigC-OA at a dose of 0.1 µg/mL (Figure 6A). These compounds at the maximum concentration tested (100 µg/mL) decreased FHs 74 Int cell viability to 17.5% and 20.2%, respectively (Figure 6A). In FHs 74 Int cell cultures, the median effective concentration (EC_50_) values were calculated at 0.32 µg/mL (266 nM/mL) for dStigC-PA and 0.47 µg/mL (381 nM/mL) for dStigC-OA. Lower cytotoxicity of dStigMAs containing a carbonate linker was observed in colon and liver cell cultures. Following cell exposure to the compounds at the maximum dose, colon and liver cell proliferation and viability were reduced to 53.8% (dStigC-PA) and 59.4% (dStigC-OA) (Figure 6B), and to 56.3% (dStigC-PA) and 48.9% (dStigC-OA) (Figure 6C), respectively. In contrast, the dStigMAs possessing a succinate linker (dStigS-OA and dStigS-PA) showed no cytotoxic activity to normal human cells when lipids were added to the cell cultures at 100 µg/mL and lower concentrations (Figure 6A–C).

## 3. Discussion

Phytosterols and their fatty acid esters naturally occur in vegetable oils and fats, where they consist up to 90% of the unsaponifiable fraction. These bioactive components reduce blood cholesterol levels in humans due to blockage of cholesterol absorption. Moreover, these compounds are often added in large amounts to various food products, the last are often treated by thermal processes such as frying. Thermal processes and long-term storage of food products enriched in plant sterols and their esters caused their degradation to oxidized derivatives, dimers, oligomers, and low molecular compounds. Some of these degradation products had adverse effects on cell cultures, while low molecular weight compounds may also negatively affect sensory perception of foods. Degradation products of stigmasterol induced apoptotic death of colon cells (CCD 841 CoN), measured by fluorescence cytometric tests [17]. The impact of sterol degradation products was time and dose dependent, however, esters formed during degradation compounds manifested lower adverse effects than free compounds. Additionally, unsaturation of fatty acid promoted degradation of steryl moiety during heating of phytosterol esters [13].

The obtained structured lipids being a hybrid of monoacylglycerols with a plant sterol could be new functional components characterized higher stability than free and esterified phytosterols. The thermo-oxidized stability of phytosterol esters depended on the unsaturation level of fatty acids moiety. The polyunsaturated fatty acids promoted the degradation of esters and the formation of phytosterol oxidation products [13]. Using saturated or monounsaturated fatty acids as a part of dStigMAs can protect sterol parts from their degradation. The enrichment of food products with monounsaturated phytosteryl ester provided the lowest rate of degradation. However, this form of sterol protection is not sufficient and further development in sterol molecules is required to eliminate the formation of compounds detrimental to human health [12]. The incorporation of the phytosterol moiety into the acylglycerol structure may have the effect of protecting it from degradation, and the use of saturated or monounsaturated fatty acids should also have a positive effect on the stability of the sterol moiety. However, before thermal-oxidative stability studies could be performed, it was necessary to check whether the obtained compounds had cytotoxic properties.

Because of that cytotoxicity of synthesized dStigMAs was determined. The analysis of their biological properties showed that hybrids with carbonate linkers characterized higher cytotoxicity than compounds with succinate linkers. It is crucial for consumers′ health and safety to establish plant sterol derivatives that will be thermo-oxidative stable and to produce benign degradation products when these bioactive compounds are used to enrich food products and to control cholesterol levels in human blood and directly affect global heart problems. Before using them as food components, a more detailed analysis should be performed.

## 4. Materials and Methods

### 4.1. Materials

Stigmasterol, stigmasteryl chloroformate (StigCF), succinic anhydride, 4-(dimethylamino)pyridine (DMAP), *N*,*N*′-dicyclohexylcarbodiimide (DCC), 3-palmitoyl-*sn*-glycerol, acetic acid, ethanol-free chloroform, ethyl acetate, methanol, *n*-hexane, dichloromethane, diisopropyl ether, toluene, and pyridine were purchased from Sigma-Aldrich (Merck KGaA, Darmstadt, Germany). 1-Oleoyl-*sn*-glycerol was purchased from Carbosynth Ltd., (Compton, UK) and glyceryl heptadecanoate was acquired from Larodan (Solna, Sweden).

### 4.2. Synthesis of Distigmasterol-Modified Acylglycerols (dStigMAs)

Commercially available 3-palmitoyl-*sn*-glycerol, 1-oleoyl-*sn*-glycerol and stigmasteryl chloroformate (StigCF) were used for the synthesis of dStigMAs. Stigmasteryl hemisuccinate (StigHS) was obtained as described below.

#### 4.2.1. Stigmasteryl Hemisuccinate (StigHS)

To a solution of stigmasterol (6 g, 14.54 mmol) in dry pyridine (100 mL), succinic anhydride (4.36 g, 43.62 mmol), and DMAP (1.77 g, 14.54 mmol) were added. The reaction mixture was stirred at 60 °C for 6 h, and then at r.t. overnight. When stigmasterol reacted completely (TLC), the reaction mixture was washed with 10% HCl solution (250 mL) and extracted with ethyl acetate (3 × 100 mL). The organic layer was washed with saturated NaCl solution (3 × 100 mL), dried over MgSO_4_, filtered, and concentrated in vacuo. The crude product was purified by recrystallization from methanol at 4 °C.

#### 4.2.2. General Procedure for the Preparation of Distigmasterylsuccinoyl-Acyl-*sn*-Glycerol (dStigS-PA and dStigS-OA)

To a solution of the corresponding monoacylglycerol (3-palmitoyl-*sn*-glycerol or 1-oleoyl-*sn*-glycerol; 0.5 g,) and stigmasteryl hemisuccinate (StigHS, 2.6 equiv.) in ethanol-free chloroform (30 mL), DMAP (2.6 equiv.) and DCC (2.6 equiv.) were added. After 24 h of stirring at 20 °C, the reaction was monitored using TLC. The mixture was filtered and the filtrate was diluted with 100 mL of chloroform and washed with 100 mL of 0.5 M HCl. The organic layer was washed with brine, dried over anhydrous MgSO_4_, and concentrated in vacuo. The crude 1,2-distigmasterylsuccinoyl-3-palmitoyl-*sn*-glycerol (dStigS-PA) and 2,3-distigmasterylsuccinoyl-1-oleoyl-*sn*-glycerol (dStigS-OA) were purified by column chromatography (hexane to hexane:EtOAc 9:1, *v/v*).

#### 4.2.3. General Procedure for the Preparation of Distigmasterylcarbonoyl-Acyl-*sn*-Glycerol (dStigC-PA and dStigC-OA)

To a solution of the corresponding monoacylglycerol (3-palmitoyl-*sn*-glycerol or 1-oleoyl-*sn*-glycerol; 0.5 g,) and DMAP (2.6 equiv.) in ethanol-free chloroform (15 mL), the solution of stigmasteryl-chloroformate (StigCF, 2.6 equiv.) in ethanol-free chloroform (10 mL) was added dropwise. The reaction mixture was stirred at 20 °C. When the reaction was finished (24 h, TLC), the solvent was evaporated and the crude 1,2-distigmasterylcarbonoyl-3-palmitoyl-*sn*-glycerol (dStigC-PA) and 2,3-distigmasterylcarbonoyl-1-oleoyl-*sn*-glycerol (dStigC-OA) were purified by column chromatography (hexane to hexane:EtOAc 9:1, *v/v*).

### 4.3. Identification of dStigMAs

TLC analyses used to monitor the progress of reactions were performed on 0.2 mm silica gel 60 F254 plates (Merck KGaA, Darmstadt, Germany) with a mixture of hexane and ethyl acetate or chloroform, methanol, and acetic acid in various ratios. Primuline spray (0.05% in acetone:water, 80:20, *v/v*) was used as a visualization reagent. Visualization was determined using UV light (λ = 254 and 365 nm). Compounds were also detected by spraying the plates with an H_2_SO_4_/CH_3_OH mixture (1:1, *v/v*), followed by heating to 120–200 °C. Products were purified using chromatography on silica gel columns (Puriflash silica HP 50 μm, Interchim, Montluçon, France). NMR spectra (^1^H, ^13^C, DEPT-135, COSY, HMQC, HMBC) were recorded on a Jeol 400 MHz Year Hold Magnet spectrometer (Jeol Ltd., Tokyo, Japan). Chemical shifts were referenced to the residual solvent signal (CDCl_3_, 99.8 atom % D, δH = 7.26, δC = 77.00). ATR-IR spectra were collected using a Nicolet iS10 spectrometer (Thermo Fisher Scientific, Waltham, MA USA). Spectra were obtained from 4000 cm^−1^ to 520 cm^−1^ at 32 scans, with a spectral resolution of 4 cm^−1^ and a blank window for background. HRMS spectra were recorded using the ESI technique on an ESI-Q-TOF Premier XE spectrometer (Waters Corp., Milford, MA, USA). Melting points (m.p., uncorrected) were determined on a Boetius apparatus (Nagema, Dresden, Germany).

### 4.4. Cytotoxicity

The cytotoxicity of dStigMAs was analyzed at the maximum concentration of 100 µg/mL, which corresponded to doses of 83 µM of dStigC-PA, 81 µM of dStigC-OA, 76 µM of dStigS-PA, and 75 µM of dStigS-OA. The minimum doses tested were 10,000-fold lower. Cell viability and metabolic activity were assessed using the MTT test following the procedure described by Olejnik et al. [18].

## 5. Conclusions

Four new structured acylglycerols containing two stigmasterol molecules and one molecule of fatty acid were synthesized. Using two different stigmasterol linkers allowed the obtainment of compounds with different chemical structures and biological properties. The dStigMAs containing a carbonate linker showed potent cytotoxicity to all of the analyzed cells whereas the dStigMAs bonded by a succinate linker (dStigS-OA and dStigS-PA) showed no cytotoxic activity against normal human cells when lipids were added to the cell cultures at 100 µg/mL and lower concentrations. The use of these compounds as functional food additives requires further studies on their thermal and oxidative stability, however, they may become a safe source of phytosterols for humans.

## Data Availability

Repository of Centre for Open Science.

## Sample Availability

Samples of these compounds are available from the authors.
